# Multifunctional hydrogels: advanced therapeutic tools for osteochondral regeneration

**DOI:** 10.1186/s40824-023-00411-9

**Published:** 2023-08-04

**Authors:** Wenqian Zhang, Kangkang Zha, Weixian Hu, Yuan Xiong, Samuel Knoedler, Doha Obed, Adriana C. Panayi, Ze Lin, Faqi Cao, Bobin Mi, Guohui Liu

**Affiliations:** 1 Department of Orthopaedics, Union Hospital, Tongji Medical College, Huazhong University of Science and Technology, Wuhan, 430022 China; 2 Hubei Province Key Laboratory of Oral and Maxillofacial Development and Regeneration, Wuhan, 430022 China; 3 Division of Plastic Surgery, Brigham and Women’s Hospital, Harvard Medical School, Boston, MA 02152 USA; 4 Department of Plastic, Aesthetic, Hand and Reconstructive Surgery, Hannover Medical School, Hannover, Germany; 5 Department of Hand, Plastic and Reconstructive Surgery, Microsurgery, Burn Center, BG Trauma Center Ludwigshafen, University of Heidelberg, 67071 Ludwigshafen/Rhine, Germany; 6 School of Chemistry, Chemical Engineering and Biotechnology, Nanyang Technological University, 21 Nanyang Link, Singapore, 637371 Singapore

**Keywords:** Osteoarthritis, Osteochondral regeneration, Hydrogel, Functionalization

## Abstract

Various joint pathologies such as osteochondritis dissecans, osteonecrosis, rheumatic disease, and trauma, may result in severe damage of articular cartilage and other joint structures, ranging from focal defects to osteoarthritis (OA). The osteochondral unit is one of the critical actors in this pathophysiological process. New approaches and applications in tissue engineering and regenerative medicine continue to drive the development of OA treatment. Hydrogel scaffolds, a component of tissue engineering, play an indispensable role in osteochondral regeneration. In this review, tissue engineering strategies regarding osteochondral regeneration were highlighted and summarized. The application of hydrogels for osteochondral regeneration within the last five years was evaluated with an emphasis on functionalized physical and chemical properties of hydrogel scaffolds, functionalized delivery hydrogel scaffolds as well as functionalized intelligent response hydrogel scaffolds. Lastly, to serve as guidance for future efforts in the creation of bioinspired hydrogel scaffolds, a succinct summary and new views for specific mechanisms, applications, and existing limitations of the newly designed functionalized hydrogel scaffolds were offered.

**Graphical Abstract:**

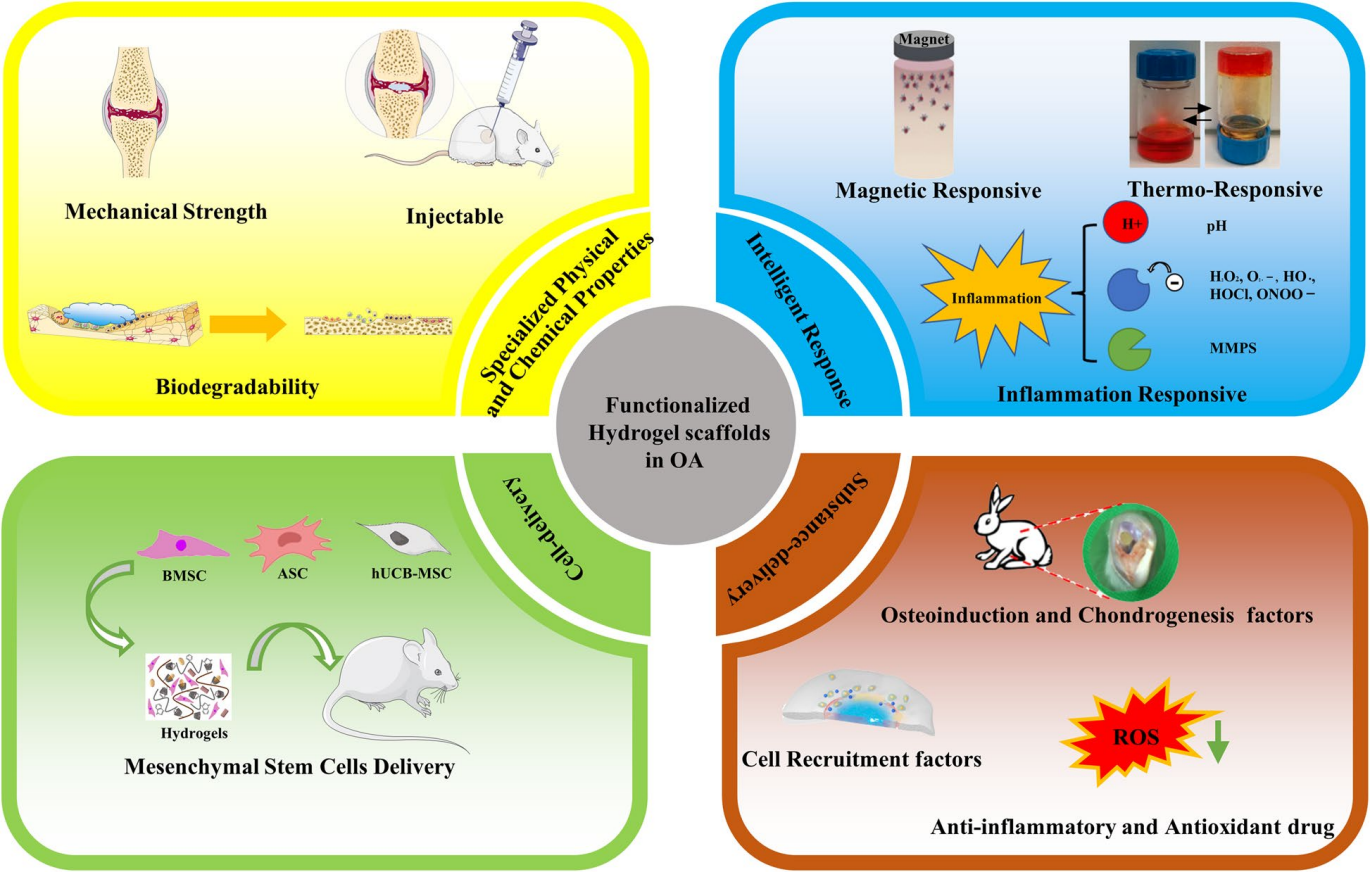

## Introduction

Osteoarthritis (OA) is the most common, frequent, and symptomatic health problem for middle-aged and elderly people. The term osteoarthritis refers to a degenerative joint disorder characterized by lesions in articular cartilage and/or subchondral bone, severe joint pain, and loss of joint function [1]. The main feature of OA is the degeneration of the cartilage matrix, further resulting in tissue lesions, which can be located deep in the osteochondral junction at later stages of the disease (osteochondral defect). Due to the avascular, less cellular, and poor regenerative nature of cartilage, the cartilage is difficult to self-heal once damaged [2]. If cartilage damages remain untreated, joints will gradually and irrevocably deteriorate, resulting in severe osteoarthritis and eventually disability [3].

Generally, surgical measures are needed to treat OA due to the poor self-repair capacity of cartilage [4]. Current therapies, concerning microfracture [5], autologous chondrocyte implantation (ACI) [6], osteotomy [7], and joint replacement [8], mainly focusing on the articular cartilage tissue, may result in inferior fibrocartilage or sometimes be poorly integrated with the subchondral bone and eventually lead to undesirable fibrocartilage formation, or poor long-term outcomes [2, 9].

Tissue engineering approaches, which aim to develop biomimetic tissue substitutes ranging from single-layered/single-component entities to bilayered/multicomponent osteochondral mimetic constructs [10, 11], offer strategies to reconstruct the osteochondral interface and repair osteochondral defects. Hydrogels, consisting of natural or synthetic hydrophilic polymer chains interconnected at the crosslinking point, demonstrate their promise in the field of regenerative medicine for their excellent qualities in biophysical and biochemical properties such as the matrix mechanics, degradability, microstructure, cell adhesion, and cell–cell interactions [12, 13]. This makes them attractive biomaterials for osteochondral tissue engineering. With the development of tissue engineering, the function of hydrogel has also changed from a single physical coverage or a single function to a combination of multiple functions now and shows a trend toward further intelligence. However, a comprehensive review of functional hydrogels in osteochondral regeneration has not been reported to date.

In this review, we summarize the tissue engineering strategies for osteochondral regeneration as well as the application of functionalized hydrogel scaffolds in the past five years. Firstly, we discuss the structure of the osteochondral unit. Further, an overview of current approaches to functionalize hydrogel scaffolds with the aim to achieve specialized physical and chemical properties, delivery ability, and intelligent response-ability is presented in this review. In the end, we provide an overview of current progress in osteochondral regeneration using functionalized hydrogel scaffolds followed by a summary and outlook on future perspectives of hydrogel scaffolds.

## Structure of the osteochondral unit and tissue engineering strategies for osteochondral regeneration

Osteochondral regeneration has always been a main challenge because of the structure and properties of the osteochondral unit. As an integrated and functional entity, the osteochondral unit, consisting of subchondral bone tissue, articular cartilage, and osteochondral interface, is anisotropic, with spatially varying compositional, structural, and functional properties [14].

Anatomically, the subchondral bone tissue consists of the subchondral cortical bone and subchondral cancellous (or trabecular) bone [15]. In the presence of multiple cell types and sufficient vasculature, subchondral bone tissue shows innate repair and regenerative abilities. Although the degradation of articular cartilage has been universally recognized as the primary hallmark of osteoarthritis, histopathological and microstructural changes of the subchondral bone are currently attracting increasing attention in the progression and pathogenesis of osteoarthritis [16].

Articular cartilage is located on the surface of movable joints, which is superficially lubricated and serves as the cushion to lower the friction between adjacent bones, transmit the mechanical loads into the deep subchondral bone plate as well as facilitate bone movement [16]. From outward to deeper levels, cartilage can be categorized into a superficial zone, middle zone, deep zone, and calcified cartilage zone based on the unique microstructure and composition of nanoscaled collagen fibers and microsized cartilage cell capsules in each zone [17]. The common characteristic of the abovementioned zones is that each zone remains to be a translucent elastic tissue without blood vessels, lymphatic vessels, or neural tubes, exhibiting limited innate self-healing ability [18]. Unlike bone regeneration, cartilage regeneration remains challenging [2].

Besides, emerging insights have been drawn into the osteochondral interface, the mineralized osteochondral interface region between the hyaline cartilage and subchondral bone. The native osteochondral interface consists of a layer of calcified cartilage, which maintains the efficient osteochondral connection where compressive, tensile, and shear forces are transmitted from the viscoelastic joint cartilage to the stiff mineralized subchondral bone [19]. Current views believe that the osteochondral interface is important in sustaining the joint’s structural integrity for its repression in curtails ectopic mineralization, bone upgrowth, and vascular invasion from the underlying bone [20, 21]. Thus, imitating the calcified cartilage zone of the osteochondral interface is a crucial aspect in cartilage tissue engineering.

## Functionalized strategies of osteochondral regeneration hydrogel scaffolds

### Functionalized physical and chemical properties hydrogel scaffolds

Biomaterials are likely to be a critical factor in the field of regenerative medicine particularly in mimicking the chemical and physical properties of the extracellular matrix (ECM). Biomaterials should be considered from several aspects including chemical complexity, stiffness and surface properties, material design, and topography [22]. Therefore, the search for optimizing of physical and chemical properties of hydrogels has become a topic of great concern. Thus, the following section will focus on functionalized physical and chemical properties of hydrogels on osteochondral regeneration concerning mechanical properties, injectable properties, and biodegradability.

#### Mechanical properties

Due to the high stress that cartilages have to bear, distinctive physical and biomechanical properties have already become one of the main concerns in osteochondral tissue engineering's complexity [23]. The osteochondral ECM is characterized by gradual changes in structure, mechanics as well as composition. Regarding the mechanics, the compressive modulus of ECM diminishes from the osseous to the chondral face [24]. The compressive modulus of trabecular bone ranges from 4.4 to 229 MPa [25], whereas the modulus of articular cartilage varies from 1.36 to 39.2 MPa [26]. Ameliorating biomechanical properties of osteochondral defect sites has been proven to accelerate osteochondral regeneration due to the tide mechanical anchoring, the combination of subchondral bone tissue as well as the relatively stable microenvironment for tissue repairing [27]. Hydrogels, based on biopolymers, exhibit many advantages in osteochondral tissue engineering. However, the low mechanical properties remain to be a big concern that limit their applications [28]. Biomacromolecules with weak mechanical properties cannot satisfy the stringent requirement for load-bearing as bioscaffolds. To solve this problem, several current studies have focused on the functionalization of hydrogel scaffolds that allow for stiff compression resistance, thus providing a stable mechanical support to form a real connection with the subchondral bone and accelerate osteochondral regeneration.

**Fig. 1:**
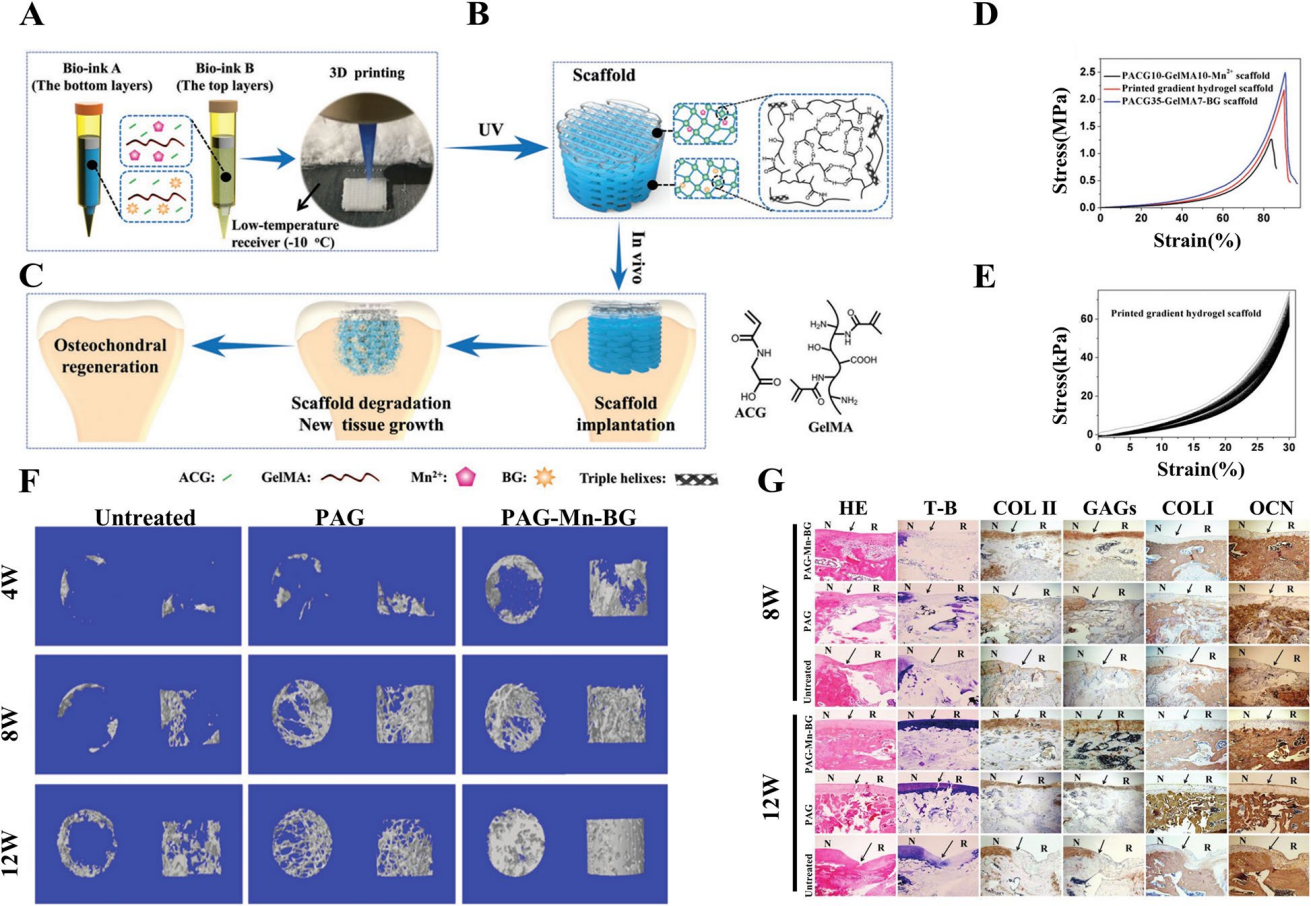
3D-Printed Biodegradable High-Strength Supramolecular Polymer Reinforced-Gelatin Hydrogel Scaffolds used in Osteochondral Regeneration. **A** The compositions of bioink A and bioink B, and 3D-bioprinting method of the biohybrid gradient scaffolds assisted with a low-temperature receiver. **B** Formation of stable hydrogel scaffold after UV light-initiated polymerization and main hydrogen bonding interactions in the PACG-GelMA network; (**C**) The repair of osteochondral defects treated with the biohybrid gradient PACG-GelMA hydrogel scaffold with Mn2 + and BG being respectively loaded on the top layers and bottom layers in animal experiment. **D** Compressive stress–strain curves of the printed hydrogel scaffolds; (**E**) Cyclic compressive stress–strain curves for the printed gradient scaffold. The cycle numbers were set as 100. **F** Characteristic 3D reconstruction images of micro-CT analysis of the repaired subchondral bone at 4, 8, and 12 weeks in different groups. **G** HE, toluidine blue (T-B) staining, and immune histological staining for Coll II, GAGs, COL I, and OCN. Copyright 2019, Wiley

Recently, a multitude of multifunctional hydrogel scaffolds that utilize this strategy to promote osteochondral regeneration was fabricated. Gao et al. [29] constructed a herein strengthened hydrogel composed of cleavable poly (N-acryloyl 2-glycine) (PACG) and methacrylated gelatin (GelMA) (PACG-GelMA) through photo-initiated polymerization (Fig. 1A, B, C). With the introduction of the biodegradable high-strength supramolecular polymer herein and hydrogen bond-strengthened PACG, they functioned the hydrogel with high tensile strength (up to 1.1 MPa), outstanding compressive strength (up to 12.4 MPa), large Young's modulus (up to 320 kPa), and high compression modulus (up to 837 kPa) (Fig. 1D, E). All these changes have increased the mechanical strength of gelatin hydrogel. Ultimately, this mechanically strengthened hydrogel scaffold not only enhanced the repair of articular cartilage but also enhanced new subchondral bone filling in the entire defect area, which illustrated its potential application as an implant for osteochondral regeneration (Fig. 1F, G).

In addition, to achieve the mechanical integration of cartilage and subchondral bone, Radhakrishnan et al. [30] developed an injectable semi-interpenetrating network hydrogel construct with chondroitin sulfate nanoparticles (ChS-NPs) and nanohydroxyapatite (nHA) (∼30–90 nm) in chondral and subchondral hydrogel zones respectively. The anisotropic construct organized with smooth transitional gradation in composition, microarchitecture, mechanical as well as biological properties was designed to mimic native interfacial tissue that regenerates and restores functional osteochondral tissue in degenerated osteoarthritis.

Despite the significant effect of mechanical properties on osteochondral regeneration, various issues still need to be considered prior to large-scale clinical application. The biocompatibility, biodegradability as well as cytotoxicity of mechanical functionalized hydrogel scaffolds in vivo need to be further explored due to their long-term presence in vivo. Besides, the combination of the present mechanically strengthened system cell printing also needs to be further explored.

#### Injectable properties

Injectable hydrogels are specialized hydrogels that can be implanted in the desired area or tissue through minimally invasive techniques. Owing to their mechanical properties, injectable hydrogels have been considered as optimal candidates for osteochondral regeneration [31]. In order to be injectable, a hydrogel is required to be liquid before and during the injection, whereas it must quickly jellify after injection to form a solid and self-standing material. In rheological terms, the elastic modulus (G′) of an injectable hydrogel must be lower than its storage modulus (G″) in order to behave as a fluid during injection, while it must form a solid (G′ > G″) once ejected [32].

Accordingly, injectable properties have been enhanced by various researchers to better suit osteochondral repairing. Chen et al. [33] hybridized alginate sodium (SA) and gellan gum (GG) with the inorganic thixotropic magnesium phosphate-based gel (TMP-BG) in the pre-crosslinking of Mg2 + to fabricate a novel hydrogel for osteochondral repairing. They introduced shear-thinning of SA-GG/TMP-BG to assure the hydrogel’s excellent injectability.

Furthermore, several injectable hydrogels are designed to functionalize viscosity while maintaining the injectable property. Chen et al. [34] fabricated an injectable adhesive hyaluronic acid (HA) hydrogel modified by aldehyde groups and methacrylate (AHAMA) on the polysaccharide backbone through an amide bond, hydrogen bond, and physical interpenetration. This AHAMA hydrogel exhibited significantly improved stability and durability within a humid environment (at least 7 days), together with higher adhesive strength (43 kPa to skin and 52 kPa to glass), thus significantly promoting integration between neo-cartilage and host tissues, and significantly improving cartilage regeneration. Li et al. [35] designed an Alg-DA/Ac-β-CD/gelatin hydrogel with the features of physical and chemical multiple crosslinking and self-healing properties. This hydrogel introduced a pre-gel state before photo-crosslinking, where decreased fluidity and increased viscosity enable the gel to remain in a semi-solid condition and make it possible for injection.

These injectable hydrogels possess good applicability, remarkable flexibility, and simple fabrication, which offer a well-suited and innovative strategy for osteochondral regeneration. However, the long-term toxicity of the biomaterial, the fusion effectiveness of hydrogels in adjacent cartilage tissue and the impact on other contributing factors to promote tissue regeneration need to be examined in the future.

#### Biodegradability

Biodegradability is crucial in tissue engineering as it enables the temporary structure and environment provided by hydrogels to be gradually replaced by ingrowing tissues and thus allow for an ameliorated repair effect without the necessity of secondary removal of the implanted hydrogels.

A multitude of multifunctional hydrogel scaffolds that optimize biodegradability to promote osteochondral regeneration were reported. Yang et al. [36] prepared a polypept(o)ide-based PAA-RGD hydrogel using a novel thiol/thioester dual-functionalized hyperbranched polypeptide P(EG3Glu-co-Cys) and maleimide-functionalized polysarcosine under biologically benign conditions, which degrades completely on day 30 after implantation, thus matching the ingrowth rate of new cartilage during the repair process for a better repair effect. Gao et al. [29] constructed a hydrogel composed of cleavable poly (N‐acryloyl 2‐glycine) (PACG) and methacrylated gelatin (GelMA) (PACG‐GelMA) by photo‐initiated polymerization, which shows tunable biodegradability by incorporating the reversible hydrogen bonds of ACG into the GelMA hydrogel system and adjusting ACG/GelMA ratios. Liao et al. [37] prepared a biphasic CAN-PAC hydrogel for osteochondral regeneration based on the density difference between the two layers through a thermally reactive, rapid cross-linking method. Due to the biodegradability, the hydrogel used as a temporary structure and environment for regeneration was gradually replaced by native-like tissue, thus acting as an effective scaffold for enhancing the regeneration of osteochondral defects.

### Functionalized delivery hydrogel scaffolds

Tissue engineering uses bionic scaffolds to simulate the cell growth microenvironment in combination with the body's self-healing ability to regulate tissue regeneration in damaged or defective tissue sites. The cell microenvironment, which is provided by tissue engineering bionics, can induce cartilage or the osteogenic differentiation of stem cells, promoting their proliferation and migration. This results in endogenous osteochondral regeneration [38]. Ideally, cartilage tissue-engineered hydrogel scaffolds should be characterized by their non-toxic, biodegradable, biocompatible, and porous properties, and should promote cell differentiation and tissue regeneration [13]. The porous structure of hydrogels makes them naturally suitable for loading a wide variety of substances and releasing them slowly at specific locations [39]. Current methods can be categorized into local delivery of exogenous cells or acellular- substances including the precise incorporation of bioactive growth factors into the target tissue, the use of cell-free scaffold biomaterials, or the mimicry of natural ECM with the use of cell-laden building scaffolds to facilitate cell organization within the ECM during reconstruction [40]. The common delivery substances are summarized (Table 1).

**Table 1:** Common nanomaterial supplementation for hydrogel scaffolds in osteochondral regeneration

Nanomaterial Supplementation Class	Name	Effects	Reference
**Metal Ions**	Manganese Ion (Mn2 +)	Enhancing the bioactivity of cartilage oligomeric matrix protein	[41]
	Magnesium Ion (Mg2 +)	Promoting the adhesion, proliferation, and differentiation of cells; inducing the deposition of bone minerals; facilitating osteogenesis	[42]
**Phytomolecules**	Honokiol (HKL)	Preventing inflammatory response and cartilage matrix degradation	[43]
	Kartogenin (KGN)	Inducing chondrogenesis of MSCs; initiating the endochondral ossification	[44]
	Chondroitin Sulfate(CS)	Anti-inflammatory effects, stimulating proteoglycan production, inhibiting cartilage cytokine production, inducing apoptosis of articular chondrocytes	[45]
**Bioceramics**	Hydroxyapatite (HAp)	Promoting bone growth	[46]
	Laponite (LAP)	Promoting chondrogenic and osteogenic differentiation of BMSCs	[47]
**Biologics**	Platelet-Rich Plasma (PRP)	Initiating and regulating cartilage healing	[48]
**Growth factors**	TGF-β1	Promoting cartilage and bone formation; chondrogenic differentiation	[49]
	TGF-β3	Regulating hyaline cartilage formation and proliferation; regulation of biosynthesis of major ECM components	[50]
	BMP-2	Enhancing osteogenesis, vascularization, and bone repair; osteogenic differentiation	[51]

#### Functionalized cell-delivery hydrogel scaffolds

Cell-laden repair, the traditional tissue engineering strategy, refers to the method of tissue reconstruction which uses biomaterials and external seeding cells to repair or replace tissue. This strategy consists primarily of combining reparative cells, such as mesenchymal stem cells (MSCs), with a biomaterial capable of supporting cell transplantation as well as their engraftment, viability, growth, differentiation, and secretory activity. The dynamic balance between the hydrogel scaffold and MSCs is well orchestrated in the regenerative process in tissue engineering. The biological behavior of the precursor cell population is under the direction of the scaffold matrix, architecture, immune cell population, remodeling, and degradation of the implanted construct [52, 53]. MSCs are an important resource for tissue repair because of their differentiation potential into a diversity of cell types including bone cells (osteoblasts), cartilage cells (chondrocytes), muscle cells (myocytes), and fat cells, as well as immunomodulation ability which helps to support the immune function by advantageously modifying the immune system's response to a threat. Over the last decades, novel therapeutic tools for osteochondral regeneration have risen from the combination of MSCs and tissue engineering biomaterials, such as hydrogel, which could serve as cell carriers [54, 55]. MSCs can be divided into auto-, allo- and xenogeneic sources. The first two sources provide an immunologically safer approach, while the latter increases the availability of MSCs enormously. This aids in the creation and reparation of skeletal tissues, such as cartilage, bone, and the fat found within bone marrows. Indeed, the use of xenogeneic MSCs in different hosts is a common tissue engineering strategy supported by numerous studies [56–58]. By far, the most common MSCs used in osteochondral regeneration tissue engineering include bone marrow-derived MSCs (BMSCs), adipose-derived stem cells (ADSCs), umbilical cord blood-derived MSCs (UMSCs) and autologous activated peripheral blood stem cells (AAPBSCs) [12].

MSCs have been universally acknowledged as a potential therapeutic method in a vast number of diseases for their ability to differentiate into diverse cell lines depending on the available niche. Furthermore, MSCs can differentiate into diverse cell types (such as osteocytes and chondrocytes), which makes them ideal candidates for the treatment of musculoskeletal lesions [59]. However, using a single suspension of MSCs may lead to poor cell retention and viability, decreasing the effectiveness of the treatment for osteochondral repair [60, 61]. Hence, the use of tissue engineering technology is promising to boost the persistence and engraftment of the implanted cells at the site of bone defects.

To meet the requirements of osteochondral repair, the fabrication of a suitable environment for the dynamic growth of stem cells in the presence of scaffolding biomaterials as well as specific growth factors have been considered as the main elements. The primary application of hydrogels is as a space-filling scaffold for the transport of cells and bioactive substances. Of note, hydrogels provide a conducive 3D microenvironment to promote the chondrogenesis of MSCs and cartilage regeneration in the osteochondral regeneration field [62, 63]. The cell-laden osteochondral repair hydrogels in the last five years are reported as follows:

Research by David Pescador et al. [64] demonstrated an elastin-like recombinamers (ELRs)-based hydrogel encapsulating MSCs to regenerate an osteochondral defect. The composition of ELRs is based on the repetition of the VPGXG pentapeptide found in natural elastin, where X (guest residue) can be any amino acid except L-proline. ELRs show thermo-sensitivity, characterized by a temperature known as the transition temperature (Tt), above which ELRs undergo a phase transition and assemble hydrophobically while they remain soluble at lower temperatures. Accordingly, this permits a homogeneous embedding of MSCs. Additionally, researchers have added the RGD cell-adhesion sequence to these ELRs, genetically altering them to perform as a vehicle for MSCs, resembling the extracellular matrix and providing a supportive environment for cells. Therefore, the ELRs-based hydrogel can be used as a successful cell carrier in which cells can differentiate and regenerate damaged tissue for osteochondral regeneration in rabbits ( New Zealand white rabbits, male, 6 months).

**Fig. 2:**
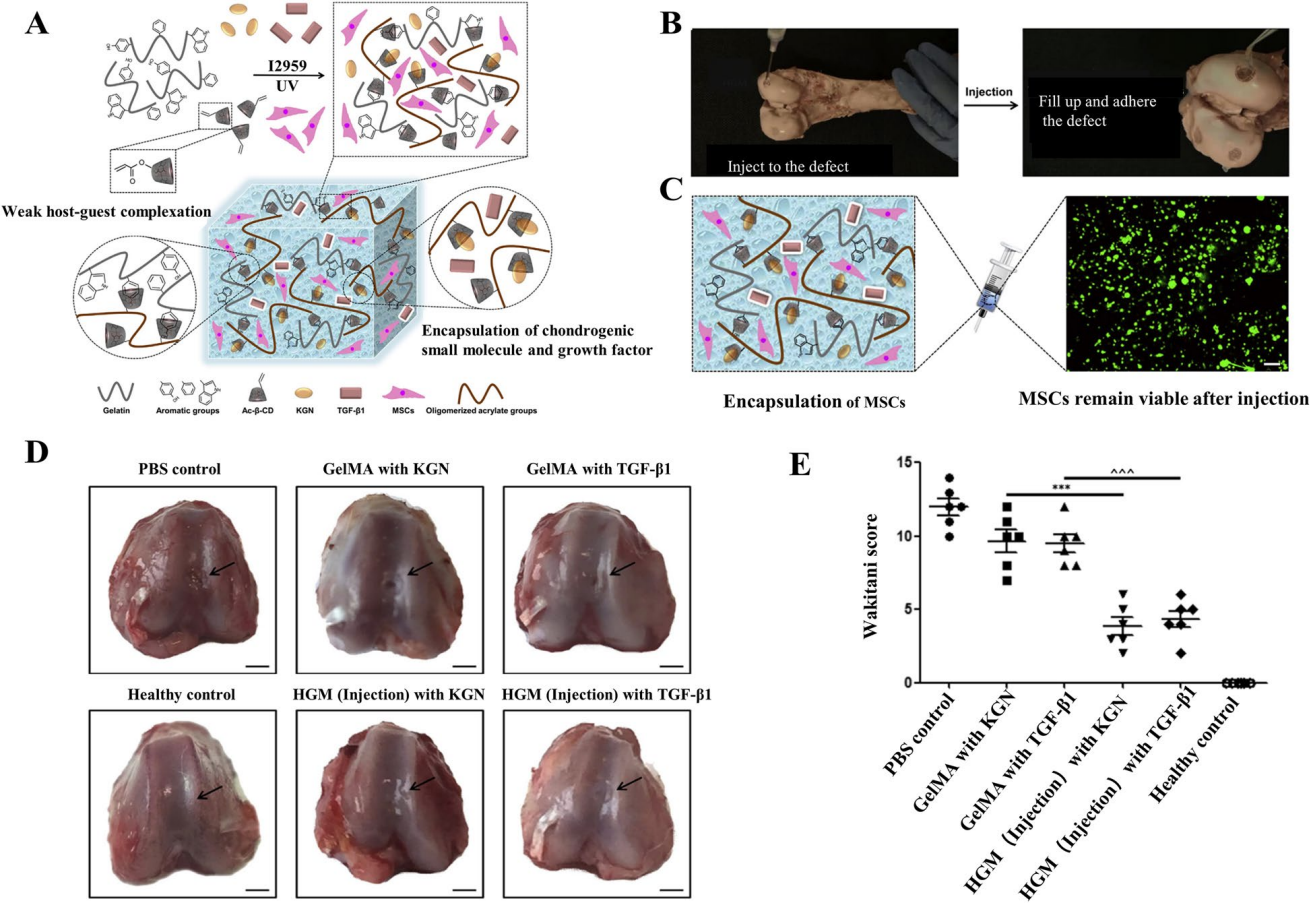
Injectable stem cell-laden gelatin HGM supramolecular hydrogels used in Osteochondral Regeneration. **A** Schematic illustration of the chondrogenic small molecules, growth factors, and encapsulation of MSCs in the injectable gelatin HGM supramolecular hydrogels. **B** The injection of pre-formed gelatin HGM supramolecular hydrogels to adhere to the cartilage defect. **C** The viability of hBMSCs in pre-formed gelatin HGM supramolecular hydrogels after injection via a G18 needle superimposed image of both calcein-AM (green, live) and ethidium bromide (red, dead) staining. Scale bar: 100 μm. **D** Macroscopic appearance of the rat knee osteochondral defect either treated with PBS or repaired by using the hydrogels loaded with chondrogenic agents at week 6 after surgery. **E** Cartilage regeneration evaluated by the Wakitani scoring system at week 6 after surgery. ∗  ∗  ∗ *P* < 0.001 vs. (GelMA with KGN); ˄˄˄*P* < 0.001 vs. (GelMA with TGF-β1). Copyright 2019, Elsevier

Jianbin Xu et al. [65] conducted the fabrication of a unique gelatin supramolecular hydrogel via a novel “Host–Guest Macromer” (HGM) approach, which stabilized by the host–guest interaction between the oligomerized acrylated β-cyclodextrins (Ac-β-CDs) and the aromatic residues of gelatin. Such gelatin HGM hydrogels showed enhanced physical and biological functionalities concerning self-healing, mechanical resilience, injectability under the gelation state, shape adapting, controlled release of hydrophobic small molecule drugs, and supporting cell infiltration [66]. In these HGM hydrogels, the hydrophobic cavity of the excess uncomplexed β-cyclodextrins (β-CDs) allows the efficient loading and the subsequent sustained release of the hydrophobic drug kartogenin (KGN), thus enhancing the chondrogenesis of the encapsulated hBMSCs (Fig. 2A). These HGM hydrogels were proven to maintain the viability of the encapsulated hBMSCs (Fig. 2B, C). In the rat osteochondral defect model, the stem cell laden HGM hydrogels worked as carrier materials of therapeutic cells that effectively promoted the regeneration of hyaline cartilage and subchondral bone (Fig. 2D, E) (SD rat, male, 4 months old).

Despite the potential benefits, the cell-seeded strategies possess various drawbacks such as relatively low cell survival rates, limited autologous cells, time/cost-intensive cell expansion procedures, as well as high risk of immune rejection [67]. Hence, several current studies are focusing on acellular-delivery hydrogel scaffolds because of the relatively low cell survival rate, scarce autologous cells, time- and money-consuming cell expansion techniques, and significant risk of immune-rejection of cell-laden hydrogels [68]. However, as the traditional and promising regenerative medicine method, efforts should still be made to optimize the cell types and medium for construct fabrication as implants for osteochondral regeneration. The application of cell-laden cartilage repair hydrogels within the last five years is summarized in Table 2.

**Table 2:** Application of functionalized cell-delivery hydrogel scaffolds within the last 5 Years

Scaffold type	Target Tissue	AT-MSCs (Implantation)	Number of AT-MSCs	Nanomaterial supplementation	Effects	Reference
Injectable ELR-based hydrogel	Hyaline cartilage	Human(Allogenic)	2.5 × 10^5^ cells/ml	Elastin-like recombinamers (ELRs)	The injectable ELR-based hydrogel led to successful regeneration of hyaline cartilage in rabbit osteochondral lesion model	[64]
Supramolecular HGM hydrogels	Hyaline cartilage; subchondral bone	Human(Allogenic)	1 × 10^7^ cells/ml	Kartogenin; TGF-β1	The injected MSC-laden HGM hydrogels led to quality neocartilage formation in the rat knee cartilage defect model	[65]
Fiber-reinforced and GF-loaded tri-layered hydrogels	Hyaline cartilage; subchondral bone	Rabbit(Allogenic)	5 × 10^6^ cells/ml	TGF-β1	The fiber-reinforced and GF-loaded tri-layered hydrogel onstruct could simultaneously facilitate the regeneration of both cartilage and subchondral bone	[69]
Injectable, self-hardening, mechanically reinforced hydrogel (Si-HPCH)	Hyaline cartilage; subchondral bone	Human(Allogenic)	1 × 10^6^ cells/ml	/	The defects filled with Si-HPCH, revealed a significant osteochondral regeneration in the canine osteochondral defect model	[70]

#### Functionalized acellular-delivery hydrogel scaffolds

New approaches and applications in tissue engineering and regenerative medicine continue to drive the development of functionalized cell-free scaffolds for osteochondral regeneration. As the drawbacks of cell-seeded strategies, these unsolved problems have motivated scientists to design functionalized scaffolds deprived of cells that are seeded in vitro (cell-free scaffolds), to assist in the recruitment of endogenous cells in vivo (tissue induction).

Cell-free repair is another tissue engineering strategy based on the mechanism of tissue induction and tissue regeneration. This strategy has drawn universal attention for its benefits with regard to harvesting, proliferating, and differentiating cells [71]. Tissue induction or tissue regeneration is an alternative way of free of external seeding cells that is contingent upon internal cells that can migrate into porous scaffolds. These cells comprise biomaterials with higher requirements as well as appropriate physical structure and chemical characteristics [72].

Within the last five years, accumulating studies have reported the application of cell-free hydrogel scaffolds in osteochondral regeneration [71, 73, 74]. In combination, this evidence infers confirmed promising effects of functionalized cell-free scaffolds in osteochondral regeneration (Table 3).

**Table 3:** Application of functionalized delivery hydrogel scaffolds within the last 5 years

Scaffold type	Description	Scaffold materials	Supplementation	Manufacturing process	Effects	Reference
**Single-layer hydrogel**	Homogeneous hydroxyapatite/alginate composite hydrogel	Alginate	Hydroxyapatite, sodium citrate	3D Bioprinting	ALG/HAP hydrogel stimulated chondrocytes to secrete calcified matrix in vitro and in vivo	[75]
	Decellularized cartilage ECM and PEGDA integrated hydrogel	Polyethylene glycol diacrylate	Honokiol, chondrocyte-derived ECM	3D Bioprinting	The decellularized cartilage PEGDA/ ECM hydrogel effectively promoted regeneration of hyaline cartilage and subchondral bone tissues in osteochondral defect model of rabbits	[76]
	3D-printed PRP-GelMA hydrogel	Gelatin methacryloyl	Platelet-rich plasma	3D Bioprinting	The 3D-printed PRP-GelMA hydrogel promoted osteochondral repair through immune regulation by M2 polarization in osteochondral defect model of rabbits	[77]
	Multifunctional polyphenol-based silk hydrogel	Silk fibroin	E7 (EPLQLKM), tannic acid	Chemical and physical crosslinking	The SF-TA-E7 hydrogels promoted enhanced regeneration of both cartilage and subchondral bone in osteochondral cylindrical defects model of rabbits	[78]
	Injectable immunomodulation-based porous chitosan microspheres/HPCH hydrogel	Porous chitosan, hydroxypropyl chitin	Kartogenin, dimethyloxallyl glycine	Chemical and physical crosslinking	The immunomodulation-based CSK-PMS hydrogel effectively created M2 macrophage microenvironment and orchestrated osteochondral regeneration in the osteochondral defect model of rats	[73]
**Multilayer hydrogel**	Biomimetic bacterial cellulose-enhanced double-network hydrogel	γ-glutamic acid, lysine, alginate, bacterial cellulose	Hydroxyapatite	Chemical and physical crosslinking	Synthesized scaffolds led to good integration between the neo-subchondral bone and the surrounding host bone in osteochondral defect model of rabbits	[79]
	Injectable BRH-CRH biphasic hydrogel	Hyaluronic acid methacryloyl, Gel methacryloyl, isocyanatoethyl acrylate-modified β- cyclodextrin	Kartogenin, melatonin	Photopolymerization	BRH-CRH biphasic hydrogel significantly promoted the simultaneous cartilage regeneration and bone regeneration to achieve osteochondral defect repair in osteochondral interface defect rabbit model	[80]
	Enzymatically crosslinked silk fibroin (SF)-Laponite (LAP) nanocomposite hydrogel	Silk fibroin	Laponite	Chemical crosslinking	The SF-LAP hydrogel promoted osteogenic and chondrogenic differentiation of BMSCs and facilitated enhanced regeneration of cartilage and subchondral bone in rabbit full- thickness osteochondral defects	[81]
	GelMA and GelMA-HAp bilayered porous hydrogel scaffolds	Gelatin methacryloyl	Hydroxyapatite	3D Bioprinting	The GelMA/GelMA-HAp bilayered porous scaffolds promoted the regeneration of articular cartilage in a rabbit trochlea model	[71]
	TGF-β loaded photo cross-linked hyaluronic acid hydrogel	Methoxy poly (ethylene glycol), poly (β-caprolactone)	Hydroxyapatite, RGD peptide, TGF-β1	Photopolymerization	The UV light-cured hyaluronic acid hydrogel containing growth factor TGF-β1 could enhance the healing of the osteochondral defect in the knees of rabbits	[82]
	Bilayered hydrogel scaffold loaded with KGN and P24 peptides	Gelatin, silk fibroin, oxidized dextran, poly (L-lactic acid), poly (Lactic-co-glycolic acid), poly(ε-caprolactone)	Kartogenin, bone morphogenetic protein—2	Chemical crosslinking	The bilayered scaffold loaded with KGN and P24 peptides significantly accelerated the regeneration of the osteochondral tissue in the rabbit knee joint model	[83]
	Integral bilayer silk scaffold consisting of a dense, smooth, biomimetic cartilage layer and a BMP-2-loaded porous layer combined with TGF-β3/Sil-MA	Methacrylated silk fibroin	TGF-β3, bone morphogenetic protein—2	Photopolymerization	The TGF-β3-loaded Sil-MA hydrogel guided new cartilage to grow towards and replace the degraded cartilage layer from the surrounding native cartilage in the early stage of knee repair	[74]
**Gradient hydrogel**	Biodegradable preprogrammed biohybrid gradient PACG-GelMA hydrogel scaffolds	Cleavable poly (N-acryloyl 2-glycine), methacrylated gelatin	Bioactive manganese ions, bioactive glass	Photopolymerization	The resultant biohybrid gradient hydrogel scaffold promoted cartilage and subchondral bone repair in rat knee osteochondral defect	[29]
	Hybridizing gellan/alginate and thixotropic magnesium phosphate-based hydrogel scaffolds	Alginate sodium, gellan gum	Magnesium	Chemical and physical crosslinking	The SA-GG/TMP-BG hydrogel scaffolds induced subchondral bone repairing and promoted the cartilage reconstruction in vivo rabbit cartilage defect model implantation	[33]
	Gradient nano-engineered in situ forming composite	Alginate, poly (vinyl alcohol)	Nanohydroxyapatite, glycosaminoglycan	Chemical crosslinking	The nanoengineered gradient hydrogel enhanced hyaline cartilage regeneration with subchondral bone formation and lateral host-tissue integration in model of rabbits	[30]

##### Functionalized anti-inflammatory drug-delivery hydrogel scaffolds

Osteochondral regeneration is a well-orchestrated process of host cell response, inflammatory immunity, as well as implant degradation in tissue engineering. Inflammation plays a crucial role in the development of osteoarthritis. Several current opinion reviews have linked the undesirable prognosis of osteoarthritis to dysregulation of M1/M2 macrophage balance [84–86]. Macrophages can be divided into M0 (resting state), M1, and M2 phenotypes. The M1-type macrophages secrete inflammatory cytokines and play a pro-inflammatory role, while the M2-type macrophages secrete anti-inflammatory cytokines for pro-tissue repair effects [87]. As the immune microenvironment plays a crucial role in bone, cartilage, and soft tissue regeneration, a disordered macrophage activation hinders the tissue regeneration process and the long-term presence of proinflammatory immune cells eventually leads to fibrous wrapping [88, 89].

In osteochondral reconstruction, macrophage phenotypes as well as cellular plasticity during the repair process can be accredited to the success of biomaterial application [90]. Targeting macrophage polarization regulation and immune modulation, several hydrogels have been designed to promote the transition from early pro-inflammatory M1 to late pro-regenerative M2 macrophages in order to ameliorate osteochondral regeneration [36, 73, 77].

**Fig. 3:**
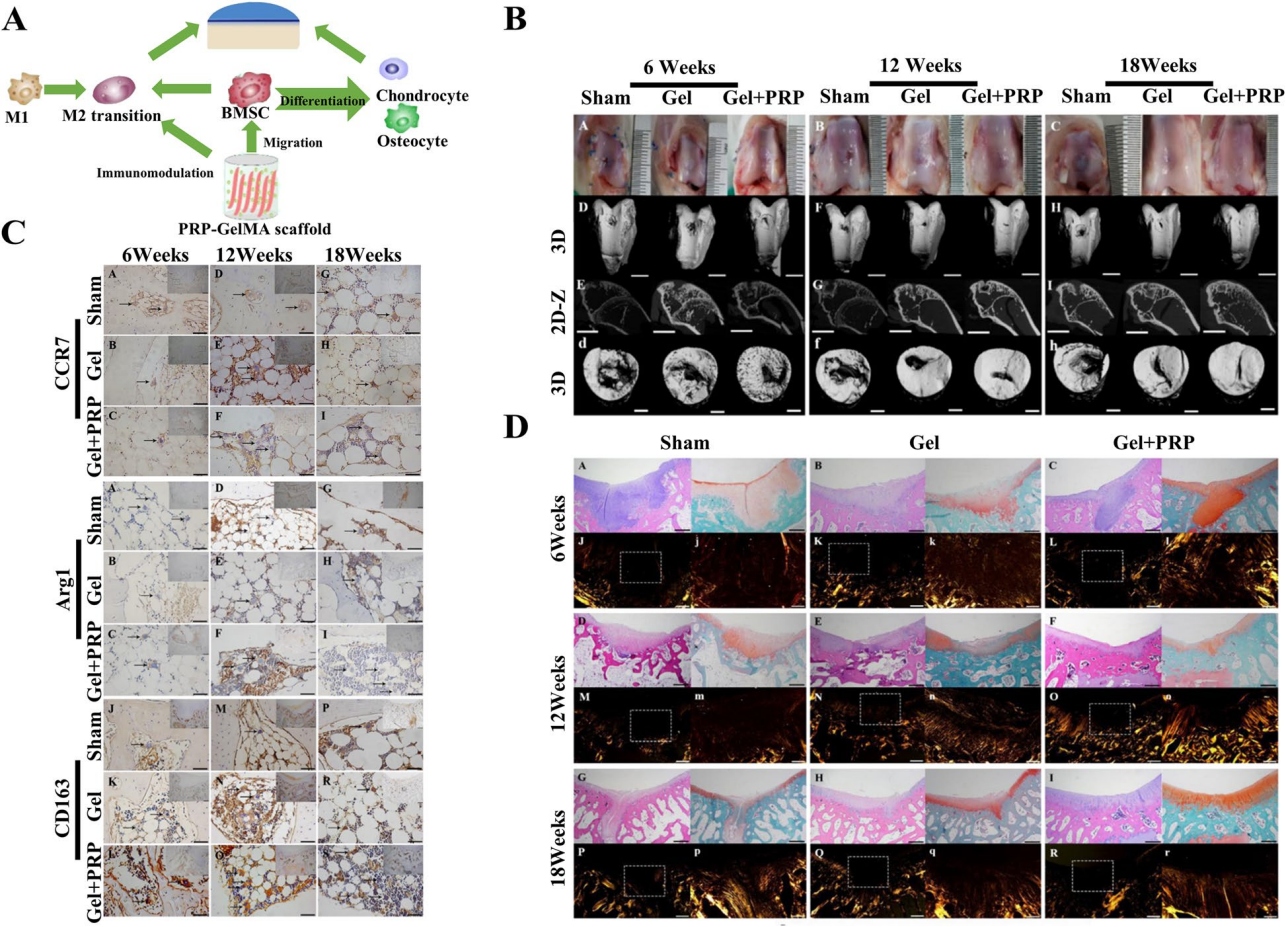
Functionalized anti-inflammatory DMOG@HPCH and CSK-PMS composite hydrogel. **A** Schematic diagram of the possible osteochondral defect-repairing mechanism of PRP-GelMA hydrogels. **B** Micro-CT and macroscopic observation of osteochondral defect repair using pure GelMA and PRP-GelMA scaffolds at 6, 12, and 18 weeks. **C** Immunohistochemical staining images of CCR7 protein, Arg 1 protein, and CD163 protein during osteochondral defect repair at 6, 12, and 18 weeks. **D** HE and Safranin-O fast green staining of osteochondral defect repair using GelMA and PRP-GelMA scaffolds at 6, 12, and 18 weeks. Copyright 2021, Elsevier

Numerous single-phase hydrogels with anti-inflammatory properties have been shown to have remarkable effects on osteochondral regeneration. For example, based on the immune regulation by M2 polarization, Jiang et al. [77] have developed a 3D-printed platelet-rich plasma (PRP)-gelatin methacryloyl (GelMA) hydrogel scaffold, which was found to play a regulatory role on BMSCs and macrophages and promote osteochondral repair in a rabbit model (Fig. 3A). Macroscopic and micro-CT observation demonstrated that smooth cartilage-like repair had integrated with the original tissue after the treatment with PRP-GelMA (Fig. 3B). (New Zealand white rabbits, male, weighing 2.5–3 kg) Histological assessment also showed promotion of cartilage repair by the PRP-GelMA scaffold through inducing local macrophage M2 polarization (Fig. 3C, D).

Additional research by Zhu et al. [76] applied a decellularized cartilage ECM and polyethylene glycol diacrylate (PEGDA) integrated hydrogel as the bio ink to fabricate a novel scaffold for osteochondral defect repair. When combined with the natural compound honokiol, this hydrogel was shown to counteract the inflammatory environment and stimulate bone and cartilage tissue regrowth in the osteochondral defect model (SD rats, male, 12 weeks).

Several muti-phasic hydrogels also have been reported to have anti-inflammatory effects. Aiming to orchestrate the immune microenvironment and providing the building-block properties to support the osteochondral reconstruction, Ji et al. [73] have developed a macrophage-modulated and injectable ‘building block’ drug delivery system comprised of dimethyloxalyl glycine (DMOG)-loaded hydroxypropyl chitin (HPCH) hydrogel (HD) together with kartogenin (KGN) conjugated chitosan (CS) PMS (CSK-PMS). This developed HD/CSK-PMS composite scaffold effectively regulated the microenvironment at the defect site and promoted cartilage regeneration in the rat OA model. (SD rats, male, weighing 300 g) These findings demonstrated that osteochondral repair efficacy can be improved by hydrogels that support macrophage M2 polarization.

##### Functionalized antioxidant drug-delivery hydrogel scaffolds

The presence of oxidative stress in the microenvironment during cartilage damage and degeneration is a significant factor in adverse and unfavorable tissue repair. In the articular cartilage damage process, the pathological acceleration of tissue metabolism and the continuous abnormal strain on the joint result in the excess activation of nicotinamide adenine dinucleotide phosphate (NADPH) oxidase in chondrocytes causing pathological production of reactive oxygen species (ROS), which leads to oxidative stress and eventually apoptosis [91, 92].

Reducing the negative effects of oxidative stress has been proposed as one of the treatment approaches to encourage the repair of osteochondral abnormalities [93]. Tissue engineering, combining biomaterials and biomolecules to provide a modified and antioxidant local microenvironment for endogenous self-repair, has emerged as a promising treatment method for osteochondral defects. Several tissue engineering hydrogels have been developed to counteract oxidative stress.

**Fig. 4:**
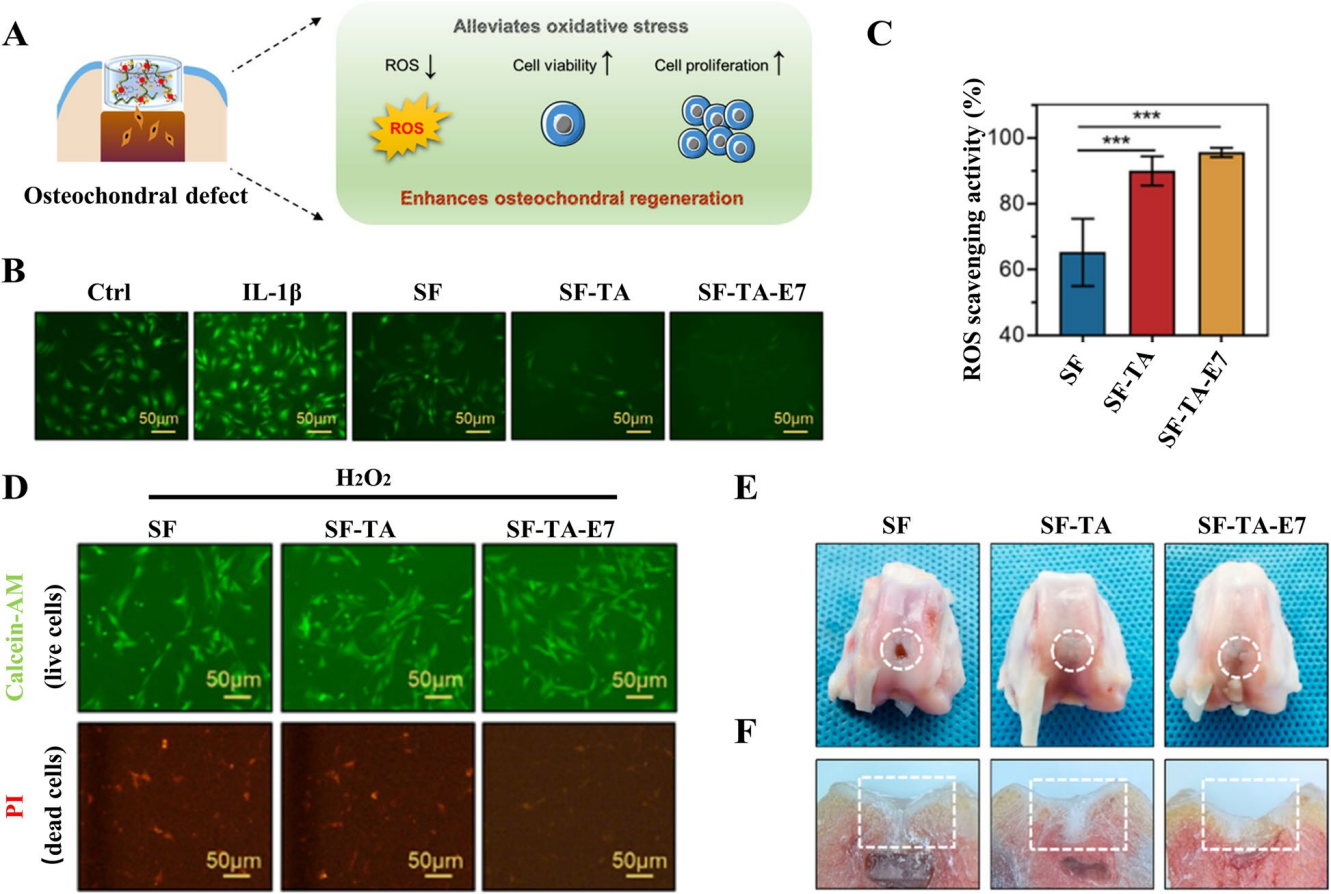
Multifunctional polyphenol-based SF-TA-E7 hydrogel alleviates oxidative stress and enhances endogenous regeneration of osteochondral defects. **A** Schematic diagram of the SF-TA hydrogel providing a supportive microenvironment to alleviate oxidative stress and enhance osteochondral regeneration. **B** Fluorescence microscope images of DCF fluorescence in BMSCs treated with various hydrogel-conditioned media. Scale bars ¼ 50 μm. **C** Intracellular ROS scavenging activity of SF, SF-TA, and SF-TA-E7 hydrogels. **D** Live/dead staining of BMSCs in H2O2-treated condition for 3 days. Scale bars ¼ 50 μm. **E** Gross morphology of joint specimens in the three groups collected at 12 weeks postoperatively. **F** Cross-sectional views of osteochondral repair at 12 weeks postoperatively. Copyright 2022, Elsevier

Several antioxidant single-phase hydrogels have been applied in osteochondral regeneration. Zhang et al. [78] have fabricated a multifunctional polyphenol-based silk fibroin (SF) hydrogel (Fig. 4A). Interacting with antioxidant tannic acid (TA), SF-TA hydrogel has been proven to eliminate ROS, thus providing a supportive microenvironment for osteochondral regeneration (Fig. 4B, C, D). In vivo experiments have also shown almost complete regeneration in cartilage surface within the SF-TA-E7 hydrogel group (Fig. 4E, F) (New Zealand white rabbits, male, weighting 2.5 kg).

##### Functionalized cell recruitment factor-delivery hydrogel scaffolds

The foundation of cell-free tissue engineering is tissue induction. In fact, numerous tissues and organs, including adipose tissue, bone marrow, and skeletal muscle, contain copious endogenous stem cells that can be drawn to defect locations for osteochondral restoration [94]. Scaffold-based techniques have advantages in osteochondral engineering since scaffolds can provide 3D microenvironments for endogenous or exogenous cells to augment cell adhesion, proliferation, migration, and differentiation [95]. Thus, the tissue engineering strategy of stimulating recruitment of endogenous stem/progenitor cells to the injury sites has drawn concern and many cell-free hydrogels to repair osteochondral defects are designed based on stem cell recruitment [96]. Some drugs and molecules have been applied to potentiate hydrogel scaffolds with the ability to attract host stem/progenitor cells. Cell-recruiting biomolecules, such as BMP-2, SDF-1α, TGF-β1, and PDGF-BB, have been widely applied to direct host stem/progenitor cell recruitment [97–99].

**Fig. 5:**
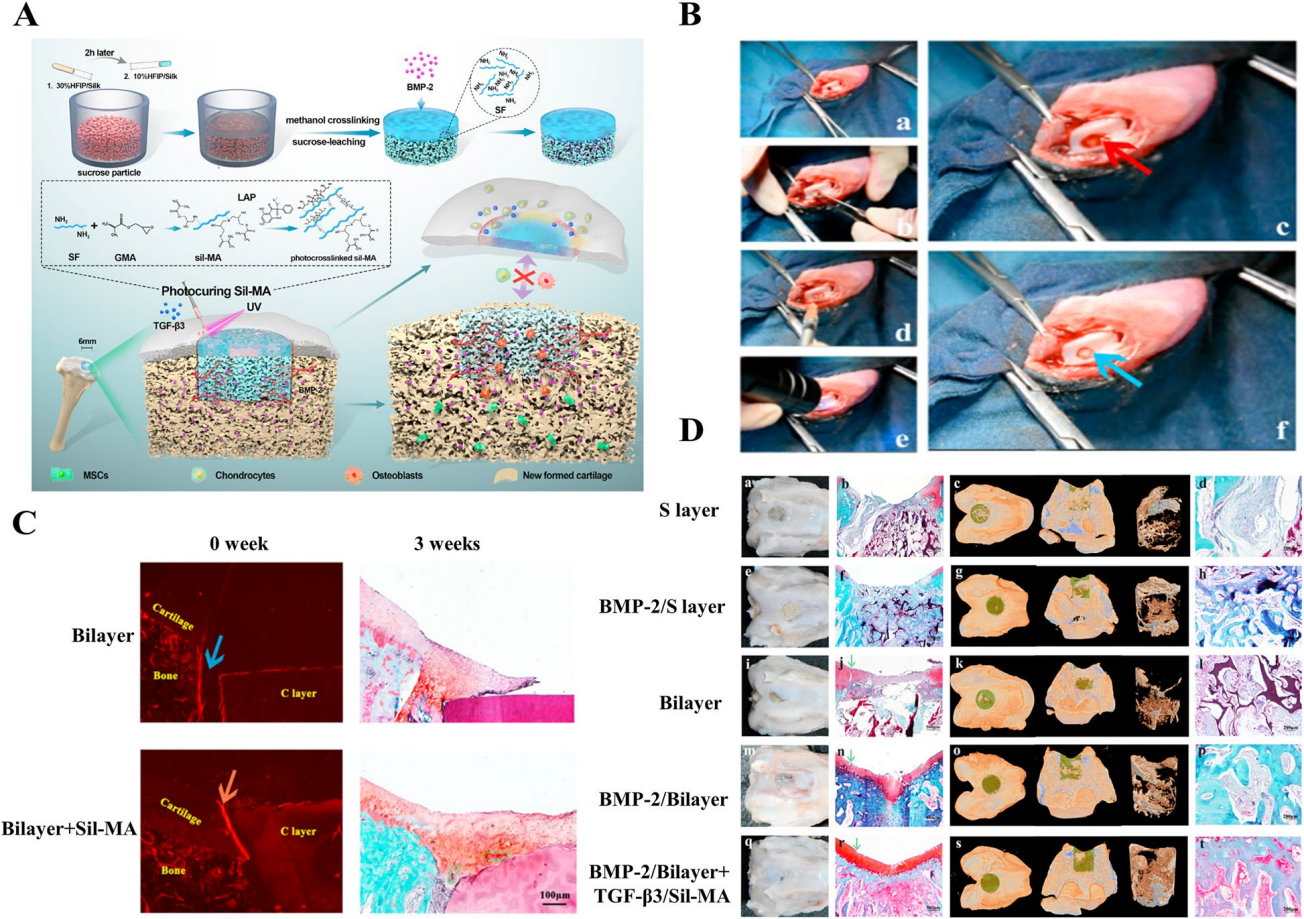
The photocurable hydrogels with TGF-β3-loaded methacrylated silk fibroin sealant promoted chondrocyte migration and differentiation. **A** Schematic illustration of the integral bi-layer silk scaffold combined with Sil-MA hydrogel in osteochondral repair through cytokines delivery and promoting of chondrocyte migration and differentiation. **B** Surgical procedures for the use of bilayer silk scaffolds combined with Sil-MA hydrogel in osteochondral repair. **C** Immediate implantation conditions at 0 weeks and repair conditions at 3 weeks were observed to evaluate the effect of the Sil-MA hydrogel. **D** Gross images, Micro-CT 3D images, and safranin-O/fast-green staining of different groups indicate lateral integration between neocartilage and adjacent cartilage. Copyright 2021, Elsevier

Several muti-phasic hydrogels have been designed based on cell recruitment. Hsieh et al. [82] have fabricated a biodegradable porous polycaprolactone (PCL) scaffold, modified by Arg-Gly-Asp (RGD) peptide grafting for cell adhesion and proliferation. Wu et al. [74] designed a bilayer silk scaffold consisting of a dense, smooth, biomimetic cartilage layer as well as a BMP-2-loaded porous layer combined with TGF-β3/Sil-MA to promote chondrocyte migration and differentiation (Fig. 5A). This TGF-β3-loaded Sil-MA hydrogel has been proven to guide new cartilage to grow towards and replace the degraded cartilage layer from the surrounding native cartilage in the early stage of knee repair (Fig. 5B, C) (New Zealand white rabbits, male, weighting 2 kg). Furthermore, consistent and white opaque tissues were observed in the regenerated area of the BMP-2/bilayer + TGF-β3/Sil-MA group after treatment for 8 weeks (Fig. 5D).

These tissue engineering hydrogels provide new insights into host cell recruitment by functionalized cell-free scaffolds that can promote osteochondral regeneration.

##### Functionalized osteoinduction and chondrogenesis factor-delivery hydrogel scaffolds

Osteoinduction refers to the ability to stimulate the differentiation of stem/progenitor cells toward osteogenic lineages in vitro [100]. Chondrogenesis refers to the process of mesenchymal stem cell (MSC) differentiation into chondrocytes [101]. As aforementioned, osteochondral regeneration is associated with osteogenic-related as well as chondrogenic-related cell production. Accordingly, cell-free hydrogel scaffolds with the capacity for osteoinduction and chondrogenesis are highly promising for osteochondral regeneration. For osteochondral tissue engineering, the ideal hydrogel scaffolds should present a favorable microenvironment for the adhesion and proliferation of stem cells (mainly BMSCs), as well as provide the inductive signals to promote osteogenic and chondrogenic differentiation of BMSCs to simultaneously regenerate articular cartilage and subchondral bone of osteochondral defects [102].

Several materials have been applied to stimulate chondrocytes to secrete calcified matrix. You et al. [75] synthesized a homogeneous ALG/HAP composite hydrogel with sodium citrate as a dispersant, and this hydrogel was shown to stimulate chondrocytes to secrete calcified matrix. Radhakrishnan et al. [30] have drafted an in situ establishing alginate/poly (vinyl alcohol) (PVA) semie-interpenetrating network (SIPN) hydrogel with layer-specific bioactive molecules (nanohydroxyapatite and glycosaminoglycan) for subchondral and cartilage layers, which showed to enhance hyaline cartilage regeneration with subchondral bone formation and lateral host-tissue integration.

**Fig. 6:**
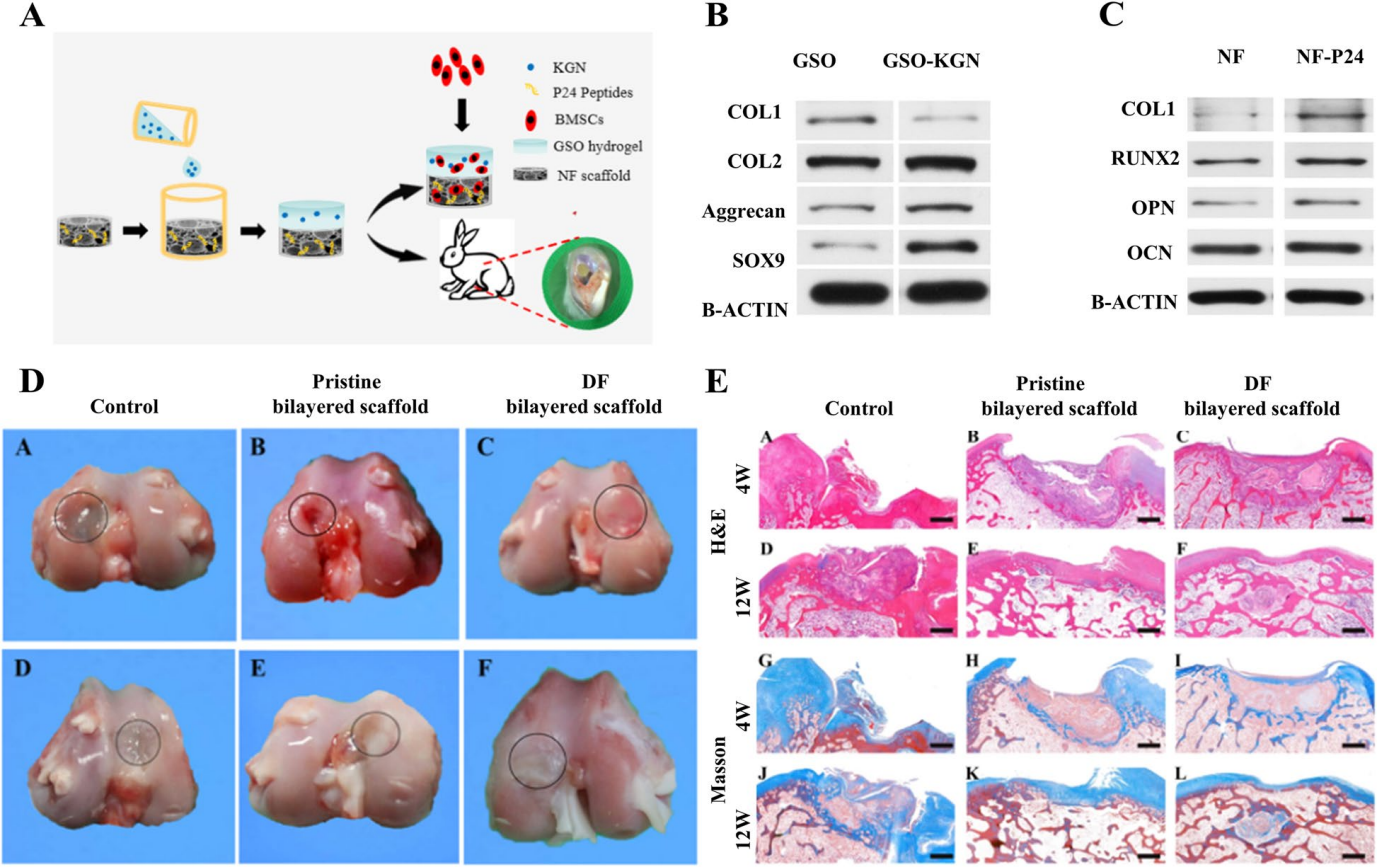
The novel drug nanobox-decorated biphasic hydrogel (named BRH-CRH) induced site-specific differentiation of MSCs into chondrocytes and osteoblasts by controllable phase/site-specific releasing kartogenin and melatonin. **A** Schematic Illustration of the Bilayered Scaffold-Loaded with KGN and BMP-2-Derived Peptides for Osteochondral Repair. **B** Cartilage relative protein expressions within BMSCs cultured on GSO hydrogels and GSO-KGN hydrogels for 21 days. **C** Bone relative protein expressions within BMSCs cultured on NF scaffolds and NF-P24 scaffolds for 21 days. **D** Macro-photographs of rabbit osteochondral defects after implanted with the control group, the pristine-bilayered scaffold group, and the DF-bilayered scaffold group for 4 and 12 weeks. **E** H&E and Masson staining images of samples after implantation for 4 and 12 weeks (scale bar = 1 mm). Copyright 2019, ACS publications

Bioactive molecules and drugs are also used in scaffolds' functionalization for both osteoinduction and chondrogenesis. Zhang et al. [81] developed a novel enzymatically crosslinked SF-LAP nanocomposite hydrogel. With the introducing of a small amount of LAP, this hydrogel encouraged osteogenic and chondrogenic differentiation of BMSCs and facilitated enhanced regeneration of cartilage and subchondral bone in rabbit full-thickness osteochondral defects. (New Zealand white rabbits, male, weighting 2.5 kg) Zheng et al. [83] constructed a bilayered scaffold containing a hydrogel-based cartilage layer and multipolymer NF scaffold-based subchondral bone layer, which induced osteogenic differentiation and bone regeneration through the incorporation of KGN and BMP-2-derived peptide (Fig. 6A). Western blotting analysis of expressions of cartilage differentiation genes showed that cartilage layer GSO-KGN hydrogels can further promote the differentiation of BMSCs into chondrocytes (Fig. 6B). The subchondral layer NF-P24 scaffolds were also confirmed to enhance in vitro osteogenic differentiation of BMSCs (Fig. 6C). Furthermore, in vivo repair evaluation indicated that the defect treated with the DF-bilayered scaffold possessed the best repair quality as almost the same staining was displayed between the regenerated tissue and surrounding tissue, and no obvious boundary was found (Fig. 6D, E) (New Zealand white rabbits, male, weighting 2.5 kg).

Hydrogels designed based on osteoinduction and chondrogenesis have been widely used in osteochondral regeneration. The above-mentioned hydrogels have demonstrated that great progress has been achieved in the development of functionalized cell-free hydrogel scaffolds for osteochondral regeneration. However, the use of bioactive molecules or materials for osteoinduction and chondrogenesis often suffers from instability, immunogenicity, high costs, and clinical side effects, which should also be taken into consideration.

### Functionalized intelligent response hydrogel scaffolds

The stimuli-responsive hydrogels are considered to be smart drug delivery systems, which enable spatiotemporal control over drug release and can effectively protect labile drugs from degradation [103, 104]. Intelligent response hydrogels can respond to a wide range of stimuli concerning external stimuli (including magnetic, temperature, ultrasound (US), photo, voltage, and mechanical friction) as well as internal stimuli (including reduction–oxidation (redox), pH, and enzymes) [105]. In this review, we summarized the application of intelligent response hydrogel scaffolds for osteochondral regeneration within the last five years (Table 4).

**Table 4:** Application of intelligent response hydrogel scaffolds within the last five years

Scaffold name	Hydrogel Material	External stimuli	Responsive shell	Bioactive agent	Target Tissue	Target cells	Effects	Reference
SPIONs	Agarose	Electromagnetic field	Superparamagnetic iron oxide nanoparticles (SPIONs)	BMP-2, TGF-β3, β-glycerophosphate	Hyaline cartilage; subchondral bone	MSCs	The vast majority of the tissue constructs developed striking white opacity at the bone end of the constructs over 28 days of using glycosylated SPIONs loaded with BMP-2 as a gradient across an hMSC-laden agarose hydrogel	[106]
Hyt@tgel	Hyaluronic acid (HA), Pluronic F-127 (F-127)	Temperature	Hyt-loading chitosan nanoparticles (HytNPs)	Hydroxytyrosol (Hyt)	Hyaline cartilage	MSCs, chondrocytes	The Hyt@tgel stimulated the regeneration of a lesioned tissue and prevent chondrocyte senescence	[107]
KGN/Dex-TSPBA@WHMs	Methacrylate gelatin (GelMA)	Reactive oxygen species (ROS)	ROS-responsive nanoparticles (KGN/Dex-TSPBA)	Kartogenin (KGN), dexamethasone (Dex)	Hyaline cartilage	MSCs, chondrocytes	It showed favorable ROS-responsive ability and enhanced chondrogenic differentiation and downregulation of pro-inflammatory factors in vitro	[108]
HA/PRP/BM hydrogel	Hyaluronic acid (HA)	pH	MnO2 (BM) nanoparticles (NPs)	Platelet-rich plasma (PRP)	Hyaline cartilage	MSCs, chondrocytes	The HA/PRP/BM hydrogel attenuated the severe oxidative stress and promoted chondrocyte proliferation. In a rat OA model, the HA/PRP/BM hydrogel suppressed cartilage matrix degradation	[109]

#### Magnetic responsive hydrogel scaffolds

The electromagnetic field (EMF) has gained popularity within tissue repairing and regenerative medicine research owing to its noninvasive properties and therapeutic potential. EMF has been reported to promote chondrogenic differentiation of MSC as well as trigger osteogenic differentiation of MSC [110, 111]. Besides, the therapeutic use of the magnetic field keeps expanding with the application of magnetic nanoparticles and magnetic-induced physical stimulation which enables the targeting of specific sites [112]. Numerous studies have proven that magnetic nanoparticles induce chondrogenic differentiation and osteogenic differentiation under the magnetic field as they bind to the cell surface [112–114]. On the other hand, adjusting the distribution of magnetic nanoparticles can also achieve the gradient drug delivery in multifunctional hydrogels. Therefore, the incorporation of magnetic nanoparticles into the hydrogels has been regarded as a promising therapeutic method in osteochondral regeneration.

**Fig. 7:**
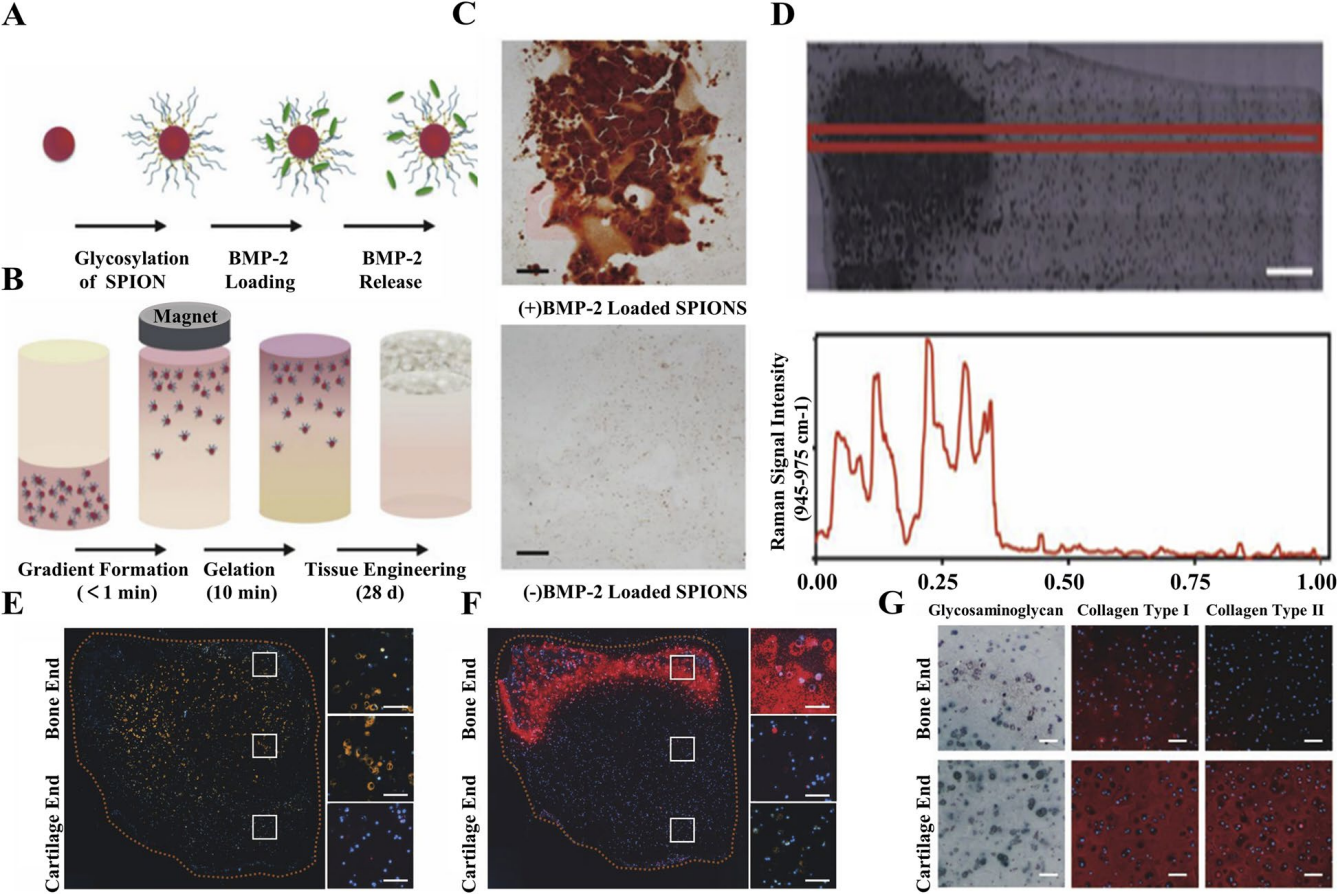
hMSC-laden hydrogel loaded with glycosylated superparamagnetic iron oxide nanoparticles (SPIONs) through an external magnetic field. **A** SPIONs are conjugated with heparin to produce a glycosylated corona that can efficiently sequester and release growth factors. **B** An external magnetic field is used to field-align glycosylated SPIONs in a hMSC-laden agarose hydrogel, which is thermally gelled and cultured for 28 days to generate robust osteochondral constructs comprising both bone and cartilage tissue. **C** An ELISA was used to detect the release of BMP-2 from glycosylated SPIONs immobilized in 1 wt% agarose, over a period of 28 days. **D** Profile of Raman intensity across the length of the osteochondral tissue construct. Scale bar ¼ 500 mm (**E**) Immunofluorescence staining of the hypertrophic protein type X collagen (orange), (**F**) Immunofluorescence staining of the key mineralization protein osteopontin (red). **G** Histological and immunofluorescence staining of key extracellular matrix proteins present in cartilage and bone revealed deposition of sulfated glycosaminoglycans (blue) and type I and II collagen (red). Scale bars ¼ 100 mm. Copyright 2018, Elsevier

Magnetic responsive hydrogel scaffolds reported within the last five years are listed as follows: Li et al. [106] used an external magnetic field to load glycosylated superparamagnetic iron oxide nanoparticles (SPIONs) into an agarose hydrogel, pre-laden with human mesenchymal stem cells (hMSCs). Thermal gelation of the hydrogel enabled us to encapsulate a stable BMP-2 gradient, which was used to spatially stimulate osteogenic gene expression and tissue mineralization over a 28-day culture (Fig. 7A,B). A stable release of BMP-2 was observed in glycosylated SPIONs immobilized in 1 wt% agarose. Through the optimization of the BMP-2 level in the glycosylated SPIONs, the local mineralization effect of the hydrogel was observed by Alizarin Red S staining (Fig. 7C). In an in vivo experiment, the application of glycosylated SPIONs into an agarose hydrogel resulted in a sharp transition in mineral content from bone to cartilage of the tidemark of the osteochondral interface-phosphate morphologies: hydroxyapatite (HAP) and β-tricalcium phosphate (β-TCP) (Fig. 7D), which act to further stimulate osteogenesis. Besides, an increased expression of the type X collagen predominantly at the interface between the bone and cartilage regions (Fig. 7E), the key marker of osteogenesis and biomineralization osteopontin presented exclusively in the bone region (Fig. 7F) as well as a higher quantity of both type I and II collagen at the cartilage end of the tissue (Fig. 7G) were observed.

Besides, Brady et al. [113] embedded magnetic nanoparticles (MNPs) and cells in layers of a trilaminar scaffold to produce an advanced smart nanocomposite hydrogel that can respond to a remote external magnetic field. Upon external magnetic stimulation, this hydrogel scaffold exhibited biochemical gradients and depth-dependent strain after 14 days in culture.

Additionally, hydrogels combined with the outer application of electromagnetic fields (EMFs) or pulsed electromagnetic fields (PEMFs) also have been widely reported in osteochondral regeneration. Li et al. [35] designed an Alg-DA/Ac-β-CD/gelatin hydrogel with PEMF treatment to enhance the therapeutic effect, which turned out to promote the quality of engineered chondrogenic constructs in vitro and facilitate chondrogenesis and cartilage repair in vivo.

Yan et al. [115] constructed a composite scaffold made of hydroxyapatite-collagen type-I (HAC) and PLGA-PEG-PLGA thermogel with EMF to stimulate bone marrow mesenchymal stem cells encapsulated in the thermogel. The combined treatment of the EMF and composite scaffold enhanced the repair of osteochondral defects in rabbits.

#### Thermo-responsive hydrogel scaffolds

Thermo-responsive hydrogel has been considered as one of the ideal drug-delivering systems. In general, to deliver drugs in locally heated tissue, the load of such materials should remain stable in normal tissues at 37 °C, but sensitive to and responsive to slight temperature changes (such as changing from hydrophilic to hydrophobic) [116]. Thermo-responsive polymers, showing sol–gel transition at 37 °C, allowing in situ hydrogel formation as well as enabling the encapsulation of drug and therapeutics at body temperature conditions, are more suitable for drug delivery [117]. The thermo-responsive polymers with in situ gel formation can be applied in osteochondral regeneration so that they can fill osteochondral defects by taking their shape, and the local injection of the polymer solution. Besides, thermos-responsive gel systems can serve as a depot after in situ gel formation, which shows timely and controlled release of drugs in vivo [118].

Previous studies have reported on the application of thermo-responsive hydrogels in osteochondral repair. To obtain a sustained and localized drug delivery at body temperature, Valentino et al. [107] constructed a localized drug delivery platform containing a combination of hydroxytyrosol-loading chitosan nanoparticles (Hyt-NPs) and an in situ forming thermosensitive hydrogel to obtain the benefits of both hydrogels and nanoparticles. This hydrogel exhibited a sol–gel transition behavior as well as a gelation time consistent with its therapeutic application. This behavior was confirmed by the measure of viscosity as a function of temperatures. Chitosan nanoparticles have been recognized as a useful drug delivery tool in OA for their ability to prolong the drug retention time. The in vitro drug release study showed a prolonged drug release of Hyt from Hyt-NPs. In an in vitro OA model, this hydrogel limited the vicious cycle typical of OA progression through the thermosensitive releasing Hyt that protected chondrocytes from ROS damage and reverted the activation of inflammatory factors.

#### Inflammation responsive hydrogel scaffolds

Inflammation is one of the main factors that contribute to the progression of osteoarthritis. In osteochondral regeneration, inflammation microenvironment, characterized by the increased expression levels of a variety of pro-inflammatory factors, higher ROS, and lower pH due to the augmented cellular ROS production and elevated metabolic activation, inhibits the repair of osteochondral tissue [119]. The modulation of inflammation in tissue microenvironment plays an important role in osteochondral repair and regeneration. To regulate inflammation, the inflammation-responsive drug release system has been considered that the drug release could be triggered under the mimicking inflammation environment and the release rate should be responsive to the inflammation degree. Owing to the characteristics of inflammation microenvironment, ROS and pH are appropriate stimulatory triggers for hydrogels specific to inflammatory diseases like OA [120].

##### ROS responsive hydrogel scaffolds

ROS-responsive nanoparticles, presented with the adequate advantage of ROS responsiveness and ROS consumption, have been extensively studied and applied in inflammatory diseases [121, 122]. Hydrogel is an ideal carrier of ROS-responsive nanoparticles as the hydrogel matrix strengthens the structural stability of the nanoparticles [123]. Meanwhile, the presence of a ROS-responsive nanoparticle leads to the elimination of intracellular ROS as well as the ROS-responsive release of drugs, thus integrating the advantages of both hydrogels and nanoparticles.

**Fig. 8:**
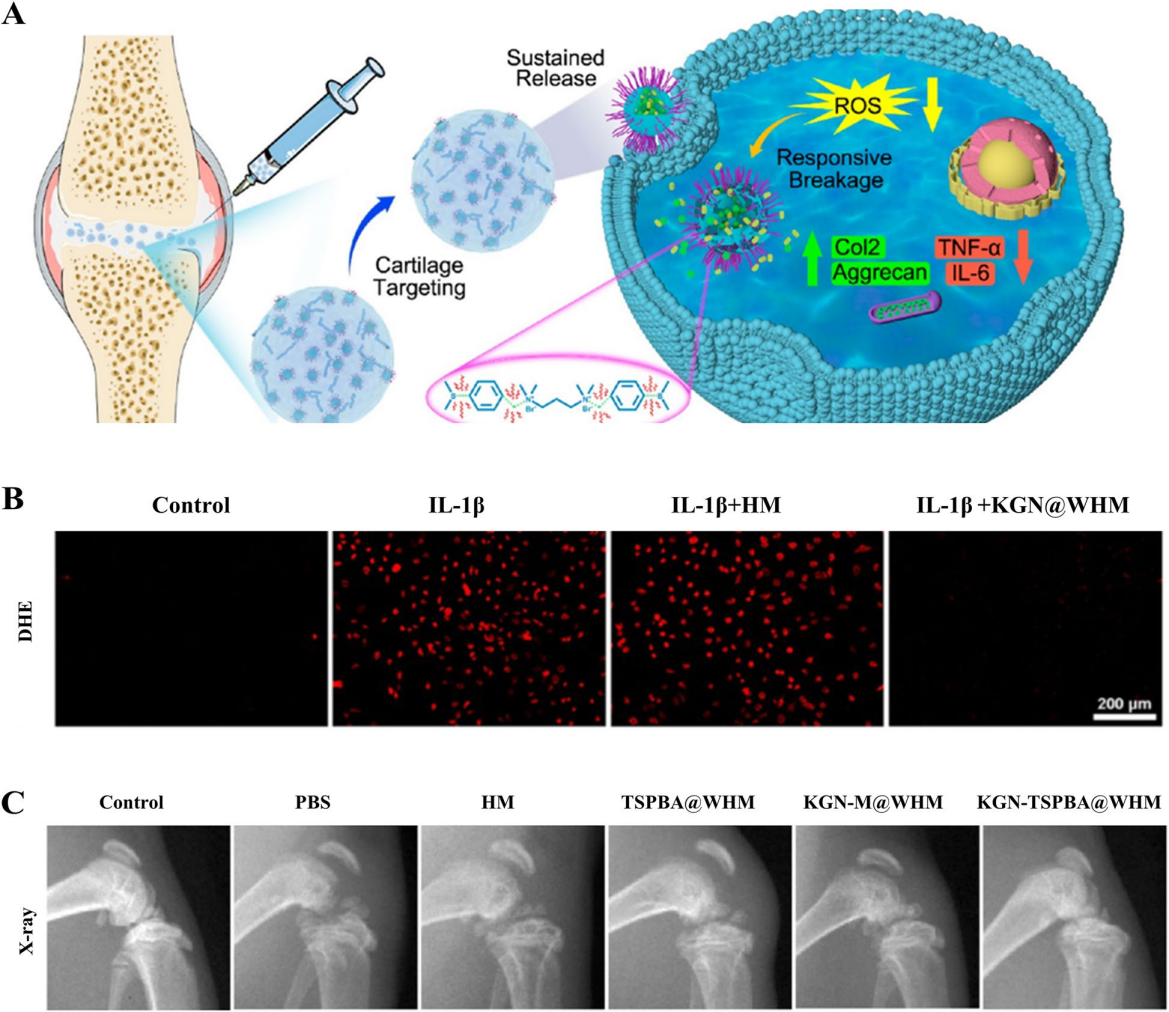
ROS-responsive injectable hydrogel microspheres (KGN/Dex-TSPBA@WHMs). **A** The KGN/Dex-TSPBA@WHMs ameliorates OA through ROS-responsive nanoparticles reacting with OA-induced intracellular ROS. **B** The dihydroethidium (DHE) staining results show the ROS-eliminating ability of KGN/Dex-TSPBA@WHMs. **C** The X-ray images of each group taken 5 weeks after the treatment. Copyright 2022, ACS publications

ROS-responsive hydrogels have been applied in osteochondral regeneration. Yu et al. [108]. designed the injectable hydrogel microspheres to anchor the ROS-responsive nanoparticles (KGN/Dex-TSPBA) and collagen II-targeting peptide WYRGRL within the matrix of the GelMA hydrogel by microfluidic technology. The responsive nanoparticles diffused from the hydrogel microspheres massively depleted the intracellular ROS and correspondingly induced the ROS-responsive release of the dual drug (Fig. 8A). Dihydroethidium (DHE) staining showed that the KGN/Dex-TSPBA@WHMs resulted in a significant decrease in the ROS level, validating that the KGN/Dex-TSPBA@WHMs could effectively mitigate oxidative stress (Fig. 8B). Besides, these hydrogel microspheres with favorable ROS-responsive ability enhanced chondrogenic differentiation as well as the downregulation of pro-inflammatory factors. As a result, KGN/Dex-TSPBA@WHMs effectively ameliorated the degradation of OA by observing the morphology of the joints (Fig. 8C). (SD rat, male, 4 months old).

##### pH-responsive hydrogel scaffolds

pH-responsive drug delivery systems have drawn universal attention due to pH regulation in inflamed tissues as pH levels differ in those compared to physiologic tissue with a pH of 7.4. This difference could be harnessed for responsive drug delivery systems to release encapsulated drugs specifically targeting these tissues [124]. To realize pH-responsive drug delivery, the formation of pH-sensitive linkages between the drug molecules and the hydrogels, such as the pH sensitivity hydrazone linkage, or the use of polymers that contain weakly acidic or basic groups in the polymer backbone. As a result, the variation in the pH level of the inflammation microenvironment will cause the drug release from hydrogels that exhibit pH-sensitive release rates [124, 125].

**Fig. 9:**
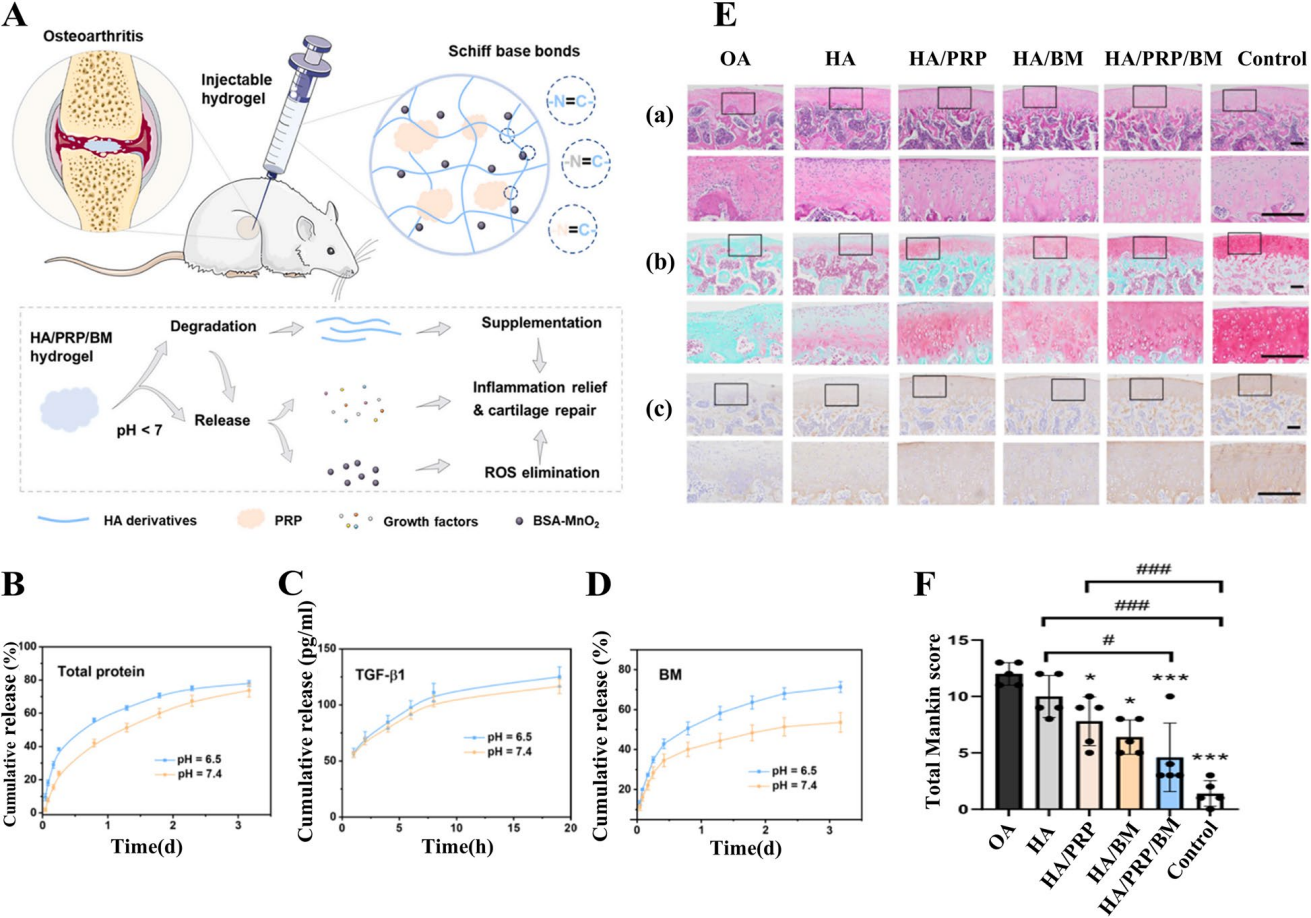
pH-responsive hyaluronic acid/platelet-rich plasma hydrogel containing MnO2 nanozymes. **A** Schematic illustration showing the injectable hydrogel of HA/PRP/BM fabricated via Schiff base reaction, and its synergetic treatment of osteoarthritis owing to visco-supplementation, ROS elimination, inflammation relief, and cartilage repair promotion. **B** The cumulative release profile of total protein from the HA/PRP hydrogel in PBS with different pH values. **V** The cumulative release profile of TGF-β1 from the HA/PRP hydrogel in PBS with different pH values. **D** The cumulative release profile of BM from HA/BM hydrogel in PBS with different pH values. **E** Representative images of HE staining, Safranin O-fast green staining, and collagen II protein immunohistochemical staining from each group. Scale bars, 200 μm. **F** Total Mankin score of articular cartilage. *n* = 5. *p < 0.05 and ***p < 0.001 versus OA group. # and ### indicate *p* < 0.05 and *p* < 0.001 between the selected groups. Copyright 2022, Elsevier

In the past several years, carbonyl-condensation reactions have emerged as versatile strategies to construct functional pH-sensitive hydrogels. The Schiff base reaction, referring to the reaction between carbonyl groups and primary amines, yields imines containing a carbon–nitrogen double bond as the product and water as the only byproduct, which is pH-responsive [126]. Zhou et al. [109] fabricated a MnO2 nanozyme-encapsulated hydrogel via dispersing bovine serum albumin (BSA)-MnO2 (BM) nanoparticles (NPs) into a hyaluronic acid (HA)/platelet-rich plasma (PRP) gel network crosslinked by Schiff base reaction (Fig. 9A). Owing to the pH-responsive properties of Schiff base bonds, the hydrogel exhibited pH-responsive release of BM NPs and growth factors (Fig. 9B, C, D). Animal experiments in a rat OA model showed that HA/PRP/BM hydrogels attenuated the severe inflammation and oxidative stress, promoted chondrocyte proliferation in vivo, and markedly suppressed cartilage matrix degradation (Fig. 9E, F) (SD rats, male, 8 weeks).

## Conclusion and prospects

This paper has provided an overview of recent developments in hydrogel scaffold functionalization techniques and applications. The review offers directions for ongoing efforts in the creation of bioinspired functionalized hydrogel scaffolds for osteochondral regeneration in addition to recent advancements.

Given the specific mechanical microenvironment of osteochondral defects, functionalized physical and chemical properties of hydrogel scaffolds are needed to provide a suitable regeneration microenvironment. Further design of hydrogel scaffolds should possess excellent mechanical properties to support newly formed tissue and stimulate osteogenic differentiation of endogenous BMSCs as well as growth and regeneration of endogenous chondrocytes. To be specific, hydrogels lack mechanical strength and are incapable to bear long-term repetitive loading in vivo [127], future directions are to developing tougher hydrogels that can withstand the long-term compression and shear in joint environment, which are promising to be achieved through combining multiple independent but interdigitating polymer networks at molecular level to construct interpenetrating network (IPN) hydrogels [128], or optimizing concentration of monomers to improve the compression modulus and the mechanical stiffness of the hydrogels [129].

Delivery of tissue-specific cells has been widely used in tissue engineering and regenerative medicine, and cytocompatible hydrogel scaffolds have been considered a promising design for tissue engineering for their structure, morphology, composition, function, and mechanics are close to the natural tissue extracellular matrix [13]. In this review, we have reviewed the reports of cell-laden osteochondral repair hydrogels within the last five years. Similar to native biological tissues, the hydrogel matrix provides a favorable microenvironment for cell function in osteochondral regeneration. Further efforts on the precise simulation and reconstruction of cartilage and osteochondral tissues are still needed. Besides, the biological, physiochemical as well as mechanical properties of the prepared composites can be tailored to patient- and tissue-specific applications. Currently, the efficiency of MSC chondrogenesis as well as cartilage tissue regeneration was not satisfactory when the combination of MSCs and existing hydrogels were applied in OA treatment [130]. Tougher hydrogels can withstand the long-term compression and shear in joint environment, but hydrogels with high stiffness are not suitable for MSC proliferation and differentiation. Thus, balancing mechanical properties, degree of hydration of the hydrogel surface, and lubricating effect should also be considered as future improvements [131].

Increasing research suggests the promising clinical application of functionalized cell-free substances delivery hydrogel scaffolds by imitating natural self-healing in vivo, which overcomes the drawbacks of cell-seeded strategies in repairing osteochondral defects. Although numerous studies have reported the application of cell-free hydrogel scaffolds within the last five years, cell-free substances delivery hydrogels face enormous challenges in moving from laboratory to clinical success. The in vivo behavior of hydrogel delivery systems is based on the data of animal models, but the curative effect of animal models for species is dependent on physiological parameters and pathologic differentiation between experimental animals and humans, which significantly disturb the accuracy of the predicted therapeutic effects in clinical trials. Besides, most of the delivery systems lack a comprehensive assessment of their local toxicity in normal tissues as well as the systemic toxicity, resulting in interruption of adverse biological interactions and intracellular signal pathways [105]. Future nanomaterial supplementations can be explored to optimize anti-inflammatory, anti-oxidative stress, cell recruitment as well as osteoinduction and chondrogenesis processes in osteochondral regeneration.

Functionalized stimuli-responsive hydrogels provide spatiotemporal control over drug release and have been proven to achieve superior targeted therapy and regeneration. Owing to the complex fabrication processes of smart stimuli responsive materials, the future exploitation of facile synthetic methodologies to replace the existing complex synthetic procedures is indispensable. Various strategies, such as external stimuli-responsive or internal microenvironment stimuli-responsive approaches, have pros and cons in practical biomedical applications [132]. Therefore, immune responses, metabolic pathways, biological distribution, as well as appropriate biodegradation rate need to be addressed in future research. Besides, the precise confirmation of optimum parameters for external stimuli and the rapid recognition of internal environmental changes are still difficult currently, which may impair the precise drug release.

In summary, current functionalized hydrogel scaffolds for osteochondral regeneration include functionalized physical and chemical properties hydrogel scaffolds, functionalized delivery hydrogel scaffolds as well as functionalized intelligent response hydrogel scaffolds. Herein, we discussed the application and drawbacks of functionalized hydrogels for osteochondral regeneration within the last five years. Given the drawbacks of the aforementioned designs, future efforts should be sustained to optimize cytological and molecular mechanisms of osteochondral regeneration as well as the biological, physiochemical, and mechanical properties of the prepared composites.

## Data Availability

Data sharing not applicable to this article.

## Abbreviations

AAPBSCs: Autologous activated peripheral blood stem cells
Ac-β-CDs: Acrylated β-cyclodextrins
ADSCs: Adipose-derived stem cells
AHAMA: Adhesive hyaluronic acid hydrogel modified by aldehyde groups and methacrylate
β-CDs: β-Cyclodextrins
BMSCs: Bone marrow-derived mesenchymal stem cells
ChS-NPs: Chondroitin sulfate nanoparticles
DMOG: Dimethyloxalyl glycine
ECM: Extracellular matrix
ELRs: Elastin-like recombinamers
GG: Gellan gum
HA: Hyaluronic acid
HAC: Hydroxyapatite-collagen type-I
HAP: Hydroxyapatite
HGM: Host–Guest Macromer
hMSCs: Human mesenchymal stem cells
HPCH: Hydroxypropyl chitin
IPN: Interpenetrating network
KGN: Kartogenin
MNPs: Magnetic nanoparticles
MSCs: Mesenchymal stem cells
NADPH: Nicotinamide adenine dinucleotide phosphate
nHA: Nanohydroxyapatite
OA: Osteoarthritis
PCL: Polycaprolactone
PEGDA: Polyethylene glycol diacrylate
PEMFs: Pulsed electromagnetic fields
PRP-GelMA: Platelet-rich plasma -gelatin methacryloyl
PVA: Poly (vinyl alcohol)
Redox: Reduction-oxidation
ROS: Reactive oxygen species
SA: Alginate sodium
SF: Silk fibroin
SIPN: Semie-interpenetrating network
SPIONs: Superparamagnetic iron oxide nanoparticles
TA: Tannic acid
TMP-BG: Thixotropic magnesium phosphate-based gel
Tt: Transition temperature
US: Ultrasound
UMSCs: Umbilical cord blood-derived mesenchymal stem cells
